# Medical Charges

**Published:** 1911-01-28

**Authors:** 


### The Hospital

A Journal of

The Medical Sciences and Hospital Administration.

New Series. No. 208, Vol. VIII. [No. 1280, Vol. XLIX.] SATURDAY, JANUARY 28, 1911.

### Medical Charges.

Once again, perhaps because this is the season of the year when doctors send out their bills, " medical charges " are under discussion; this time in the columns of our leading daily paper. There is no smoke without fire, as the saying is; and without doubt there are a good many members of the public who do feel what they believe to be legitimate grievances, both as to the fees demanded by some medical men and as to the manner of the demand. The gist of the letter which initiated this correspondence, and was published in the *Times* over the signature " Senior," may be shortly given. One complaint is of the exorbitant charges of specialists, with particular reference to surgeons; the other, of the established practice of the profession in sending out a mere statement of the amount due without giving any particulars of the items of that total.

To deal with the latter complaint first, it can well be understood that there are methodical and economical souls to whom the plan is extremely abhorrent. The answer, of course, is that any patient who demands to have an itemised account is entitled to have one, and will find no difficulty (in the great majority of cases) in getting it. But to this process there are, it must be conceded, two objections; some people who strongly desire to have such an account hesitate to ask openly for it, and some doctors would regard the request as a slur upon their honour, and might take grave umbrage when it was preferred. We do not agree that a doctor has any right to take offence when asked for the items of his account; he should be prepared to give them always. But there are some few highly sensitive men who might feel keenly that the demand savours of distrust. Nor do we think that any layman who wants such an account need hesitate to ask for it; in ninety-nine cases out of a hundred he will be cheerfully and willingly supplied. After all, the patient who takes so keen an interest in his bills is he who is most likely to pay them promptly when convinced of their fairness. It is the feeling that the doctor, or his secretary, if he keeps one, is not infallible in his accountancy, and that mistakes may be made which there is no means of checking, that produces this discontent with the old-fashioned form of account for " professional attendance."

The correspondent who has been referred to is evidently of this view. He asks why doctors should not send in their bills in detail just as lawyers do. To that question a very complete answer is given by a subsequent writer, whose identity is concealed as " Physician "; but there are other reasons as well which may be mentioned first. Solicitors, as is well known, are forbidden by law to frame their charges otherwise than by items and on certain prescribed lines; and must therefore, to make a living, charge apparently the most monstrous sums for the most trivial services, because for some of their most valuable work they are forbidden to charge at all. We may forbear to dilate upon the evidenty greater confidence which the law has in the honesty of physicians; but it must be pointed out that the truer analogy is between the doctor and the barrister, not the solicitor. The barrister charges a lump sum, and engages to do his best for his client, as does the doctor for his patient. He gives no list of the number of hours' thought devoted to his brief, or the number of questions which he asks in cross-examining hostile witnesses. Yet no one makes an outcry against barristers' fees, which are much higher than surgeons'; moreover, the latter at least does the operation for which he is paid, and never leaves it to his assistant, as a barrister often leaves a case to his junior.

As a matter of plain fact, the public would soon find out that their doctoring would cost them more under such a system. We affirm positively that medical men constantly use this method of accounting as a means of saving the pockets of those whom they know to be in straitened circumstances. A doctor, for instance, knows that if he charges anyone in a certain street, or of a certain class, less than a certain sum per visit, everyone else in that street, or that class, will demand to be treated on the same terms. So, knowing that some particular patient is less well off than his neighbours, he deliberately charges only for every other visit, or for one visit in three perhaps, and is still able to save his face and that of his patient in that he can honestly say he has not lowered his fee. No, the public stands to lose more than to gain by altering the present system; alter it, and doctors will start to charge for every letter written about the affairs of their patients, and quite properly, too. As it is, many a hard-worked doctor spends a couple of hours after his proper daily work is done in writing letters to the relatives and friends of his patients. Very rarely indeed is any charge made for this, though there is really no reason why the trouble thus entailed should not be recompensed.

Lastly, upon the subject of exorbitant fees for consultations with specialists and for operations. This point has been well dealt with in the correspondence; but once again the analogy of the barrister may be invoked. No one grumbles when a barrister refuses to accept a brief except at a high fee—and barristers' fees are much higher than any in the medical profession. Nor do barristers of eminence abate their fees to suit their clients' purses; if the latter cannot afford Mr. Silk, let them brief Mr. Stuff. Whereas consulting physicians and surgeons, save for a handful who can more than fill their time at top fees only, will always take the word of the general practitioner and reduce their fees for those in poor circumstances. If the correspondence referred to drives some of these facts into the minds of the laity, it will have done much good; and if it induces doctors to be more businesslike than many of them are, it will do wonders.

